# Suicide Attempts during Pregnancy and Postpartum: A Systematic Review and Meta-Analysis

**DOI:** 10.1007/s10995-024-03956-w

**Published:** 2024-06-29

**Authors:** Estel Gelabert, Anna Plaza, Alba Roca-Lecumberri, Alessandra Bramante, Valeria Brenna, Lluisa Garcia-Esteve, Ilaria Lega, Susana Subirà, Carolina Toscano, Anna Torres-Giménez

**Affiliations:** 1 Departament of Clinical and Health Psychology, Universitat Autònoma de Barcelona, Barcelona, Spain; 2 Unitat de Salut Mental Perinatal CLINIC-BCN, Hospital Clínic, Barcelona, Spain; 3 Unitat de Crisi i Prevenció del Suicidi, CPB-Dreta Eixample, Barcelona, Spain; 4 Società Marcè Italiana, Milan, Italy; 5 Department of Mental Health and Addiction Services, Niguarda Hospital, Milano, Italy; 6 Instituto Superiore di Sanità, Roma, Italy; 7 Psychology Research Center (CIPsi), University of Minho, Braga, Portugal

**Keywords:** Suicide attempts, Pregnancy, Postpartum, Risk factors

## Abstract

**Purpose:**

Suicide attempts (SA) during perinatal period have the potential to adversely affect a woman’s health and her developing infant. To date, little is known about perinatal SA and their risk factors. This study aimed to synthetize the evidence on risk factors of SA in pregnant and postpartum women.

**Methods:**

We systematically reviewed studies retrieved from PubMed/Medline, PsycINFO, and CINAHL, following the PRISMA guidelines for reporting. A meta-analysis was conducted only for risk factors examined in at least three distinct samples.

**Results:**

A total of ten studies were eligible for inclusion. All the studies found significant associations in regression models between perinatal SA and other variables (sociodemographic, clinical factors obstetric, neonatal, and psychosocial). The meta-analysis showed that unmarried women (pooled OR = 1.87, 95% CI = 1.26–2.78), with no higher education (pooled OR = 1.89, 95% CI = 1.31–2.74) and affected by a mood disorder (pooled OR = 11.43, 95% CI = 1.56–83.87) have a higher risk of postpartum SA; women who smoke during pregnancy (pooled OR = 3.87, 95% CI = 1.35–11.11) have a higher risk of SA in pregnancy; and women with previous suicidal behavior(pooled OR = 38.04, 95% CI = 3.36–431.17) have a higher risk of perinatal SA, whether during pregnancy or in the postpartum period. The type of sample, whether community or clinical, is a relevant moderating factor.

**Conclusion:**

Our study extends prior reviews about suicidal behaviors in women by studying perinatal suicide attempts independently, as well as it synthesized data on some sociodemographic, clinical, and obstetric/neonatal risk factors. Further studies about specific risk factors for perinatal SA are needed in order to improve early detection and intervention of women at risk.

**Supplementary Information:**

The online version contains supplementary material available at 10.1007/s10995-024-03956-w.

## Significance

Suicide attempts during pregnancy and the postpartum period pose a severe risk to the health of women and the development of their infants. Despite their importance, little is known about specific risk factors for these attempts during the perinatal period. This study is the first meta-analysis to synthesize risk factors associated with suicide attempts in pregnant and postpartum women. We found that unmarried women, those without higher education, and those with mood disorders are at higher risk for postpartum suicide attempts; women who smoke during pregnancy are at higher risk for suicide attempts during pregnancy; and those with previous suicidal behavior are at higher risk for perinatal suicide attempts. Our study extends prior reviews by independently examining perinatal suicide attempts and synthesizing data on sociodemographic, clinical, and obstetric/neonatal risk factors. Further studies on specific risk factors for perinatal suicide attempts are needed to improve early detection and intervention for women at risk.

## Introduction

Suicide is a major public health problem worldwide (World Health Organization, 2014) and an important and potentially preventable cause of maternal injury (Palladino et al., 2011). Suicide is a leading cause of death during the perinatal period (from pregnancy through the first year after childbirth) accounting for 5 to 20% of maternal deaths in high-income countries (Grigoriadis et al., 2017). In low-income and middle-income countries, the pooled prevalence was 1.00% for suicide and 5.06% for injuries (Fuhr et al., 2014). When all maternal deaths within a year after delivery are considered, suicide is one of the leading causes of maternal deaths overall (Trost et al., 2021; Diguisto et al., 2022; Lommerse et al., 2024).

Suicidality, encompassing suicidal ideation (thoughts of suicide) and suicide attempts, is considered a serious problem in perinatal women because of its impact in maternal mortality, perinatal outcomes, infant development, familial dynamics, and community welfare (Gentile, 2011; Gold et al., 2012; Shigemi et al., 2020; Zhong et al., 2019).

Half of people who completed suicide have made a previous suicide attempt (SA). It is estimated that the risk for suicide in people with previous SA is 100 times higher than the general population and 4 times higher than the people with a mental disorder (World Health Organization, 2014). In perinatal period the previous SA could also be an indicator of higher risk to complete suicide. A study conducted by Khalifeh et al. (2016) found that a quarter of perinatal women who died by suicide had self-harmed in the past 3 months and 48% had history of self-harm.

A recent meta-analysis of observational studies (Rao et al., 2021) described the pooled prevalence of SA was 680 per 100,000 (95% confidence interval 0.10–4.69%) during pregnancy and 210 per 100,000 (95% confidence interval 0.01–3.21%) during the first-year postpartum. Although this prevalence of SA is similar to the one-year prevalence of SA in the female general population reported by Borges et al. (2010), the negative impact of SA on maternal and child health outcomes, as well as the potential loss of life, must be seriously considered.

Although suicide during pregnancy and/or the postpartum period is a real public health problem, little is known about perinatal SA or their risk factors. Most of the studies examining risk factors associated with perinatal suicidality do not distinguish between suicidal ideation and SA. Identification of clinical, obstetric, neonatal and psychosocial factors associated with suicide attempts in the perinatal period may help clinicians to better identify populations at risk. Proper identification may improve prevention of SA and its consequences.

To date, no systematic review with meta-analysis synthesized specifically the risk factors of perinatal SA; thus, we conducted this systematic review and meta-analysis to examine the risk factors of SA in pregnant and postpartum women. Specifically, we hypothesize that this systematic will identify specific risk factors during the perinatal period that are associated with SA in women.

## Methods

### Eligibility Criteria

The study protocol of the systematic review was registered on PROSPERO (Protocol number: CRD42022269524). We followed the PRISMA guidelines (Liberati et al., 2009) for reporting this systematic review.

The studies of interest for this review were those that examined risk factors for SA in perinatal women. The target population was pregnant women or women up to the first year postpartum. Suicide attempts could be reported by the patients or medical reports. Quantitative empirical studies with cross-sectional, cohort or case-control designs were included. Grey literature such as theses, dissertations or conference proceedings were excluded. Case studies or reviews were also excluded. Only articles in English or Spanish were included.

### Information Sources and Search Strategies

The bibliographic search, selection of the studies and assessment of the quality were carried out independently by two researchers (EG and AT). We searched the following electronic databases: PUBMED, CINAHL, PsycINFO for peer reviewed publications. The search was with no date restriction until October 2021. Keywords included (pregnan* OR antenatal OR prenatal OR postnatal OR postpartum OR perinatal) AND (“suicidal behav*” OR “suicide attempt*” OR suicide OR “suicidal ideation”). Additionally, we searched for additional studies using Google Scholar, and screened the references lists of all eligible studies.

### Study Selection

Rayyan (https://www.rayyan.ai/), the online systematic review tool was used to manage and screen all retrieved papers. A team member was responsible for adding or amending paper records in Rayyan, as well as identifying and removing duplicates. A second independent review was given access to titles and abstracts, within Rayyan. They were able to make their own decisions as to whether to include or exclude a paper. Both reviewers were blind to other’s decisions until all titles and abstracts were reviewed. Full-text screening was also conducted independently by the same two researchers. Disagreements were resolved by discussion and consensus, and a third reviewer was consulted, if required.

### Assessment of Study Quality

The overall quality of each study was evaluated by two independent researchers using the Newcastle Ottawa Scale (NOS, Wells et al., 2021) and the Newcastle-Ottawa Scale adapted for cross-sectional studies (Herzog et al., 2013). NOS is based on a star scoring system, with a maximum score of 9 (for prospective and cross-sectional studies) and 10 scores (for case-control studies) that can be awarded to each study. Quality assessment was also checked independently, and any disagreements were resolved through discussion and consensus.

### Data Extraction and Analysis

A standardized data extraction tool was used on included studies. Extraction information include: (1) Author and publication date; (2) Country; (3) Study design and setting; (4) Suicidal attempt measure; 4) Risk factors and measures; (5) Statistical analysis; (6) Results.First, we analyzed the studies included in the review qualitatively. Only variables with a minimum of 3studies were considered candidates for meta-analysis. Although there is no consensus on the minimum number of studies needed for meta-analysis, this decision aligns with other published meta-analyses on risk factors (Xie et al., 2023). All variables were analyzed as dichotomous variables purposely. Variables were only dichotomized if the variable definitions were equivalent. The odds ratio was calculated with its 95% confidence interval. The Mantel Haenszel method with random effects model was used to compute pooled odds ratios. Heterogeneity between studies was assessed using the I-squared statistic. All analyzes were performed using STATA v.16.

## Results

**Fig. 1:**
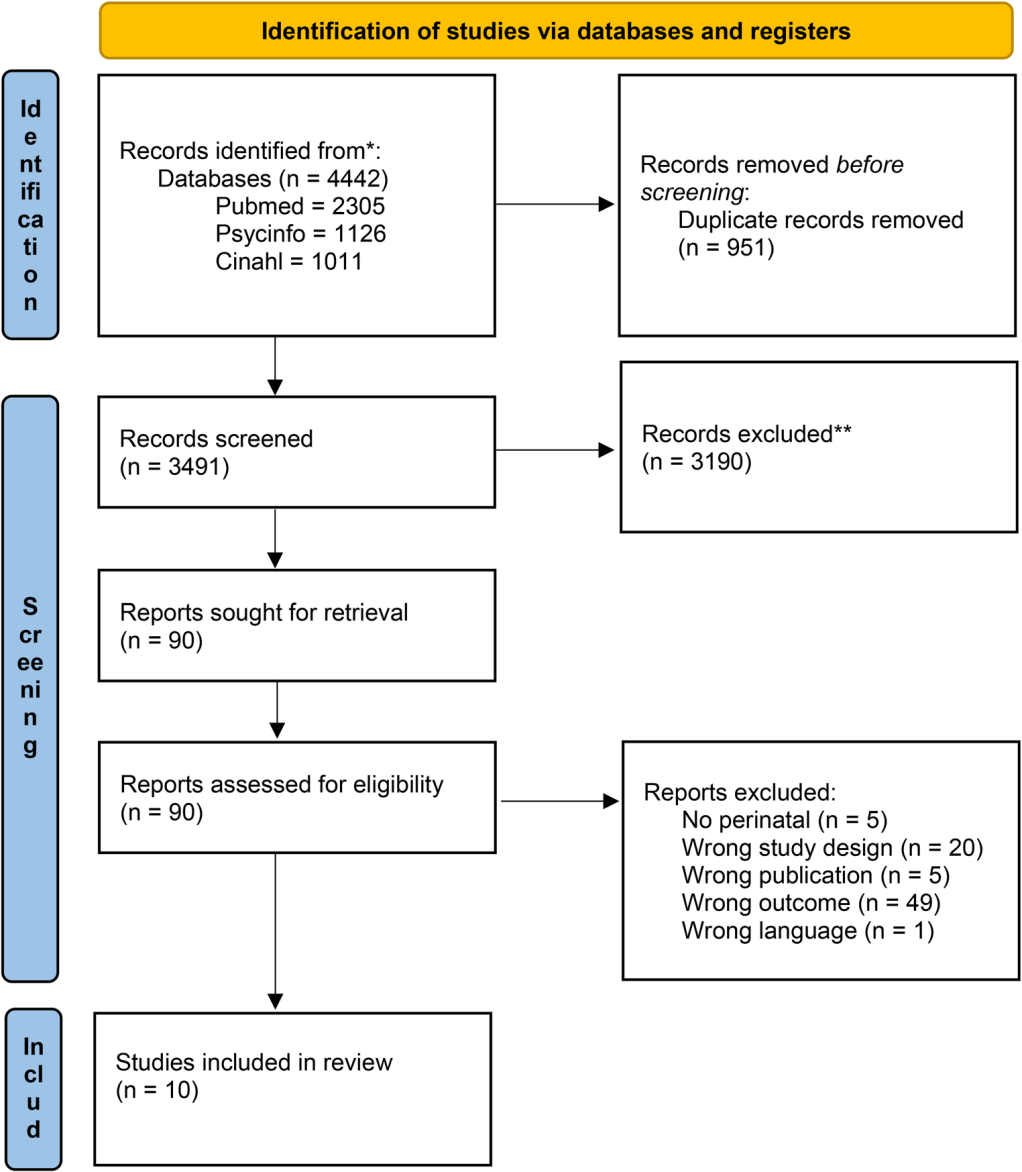
PRISMA 2020 flow diagram *Consider, if feasible to do so, reporting the number of records identified from each database or register searched (rather than the total number across all databases/registers). **If automation tools were used, indicate how many records were excluded by a human and how many were excluded by automation tools. *From*: Page MJ, McKenzie JE, Bossuyt PM, Boutron I, Hoffmann TC, Mulrow CD, et al. The PRISMA 2020 statement: an updated guideline for reporting systematic reviews. BMJ 2021;372:n71. doi: 10.1136/bmj.n71 For more information, visit: http://www.prisma-statement.org/

The PRISMA flow diagram in Fig. 1 summarizes the study selection process. The initial search yielded 4442 articles. After duplicates were excluded, the titles and abstracts of 3491 were screened, leaving 90 to be screened through full text. Of these, 80 were excluded; thus, the final sample consisted of 10 studies.

### Characteristics of the Studies

Table 1 presents the main characteristics of the studies included in this systematic review. They were conducted in 7 different countries in Europe, North America, Asia, and Africa. The most common type of study were case-control studies (*n* = 7) followed by cohort studies (*n* = 3). Almost all studies studied community population (*n* = 8) and sample size ranged from 420 to 1,269,781 subjects. Studies involving a clinical population refer to a sample of mothers with psychiatric disorder admitted to Mother Baby Units (Gressier et al., 2017), and pregnant women with psychotic or bipolar disorder (Taylor et al., 2016).

**Table 1:** Characteristics of the studies included in the systematic review

Author, Year	Country	Design	Type of sample	*N*	Period of assesment	Mesure of SA	Quality
Gressier et al., 2017	France	Case-control	Clinical	154 cases 1285 controls	Pregnancy and Postpartum	Medical records	6
Johannsen et al., 2020	Denmark	Cohort	Community	1,202,292	Postpartum	ICD codes	8
Vigod et al., 2019	Canada	Cohort	Community	1,269,781	Postpartum	ICD codes	8
Comtois et al., 2008	USA	Case-control	Community	350 cases 1420 controls	Postpartum	ICD codes	8
Weng et al., 2016	Taiwan	Case-control	Community	139 cases 1390 controls	Postpartum	ICD codes	8
Morgan et al., 1997	United Kingdom	Cohort	Community	408,000	Postpartum	N.D.	6
Schiff et al., 2006	USA	Case-control	Community	520 cases 2204 controls	Postpartum	ICD codes	7
Weng et al., 2018	Taiwan	Case-control	Community	485 cases 4850 controls	Postpartum	ICD codes	8
Taylor et al., 2016	United Kingdom	Case-control	Clinical	33 cases 387 controls	Pregnancy	Medical records	8
Belete et al., 2021	Ethiopia	Case-control	Community	20 cases 718 controls	Pregnancy	CIDI	6

SA: Suicide Attempt; ICD: International Classification of Diseases; CIDI: Composite International Diagnostic Interview. N.D.= Non defined

The measure most commonly used to assess SA was the International Statistical Classification of Diseases and Related Health Problems (ICD) codification (World Health Organization, 1966, 1978, 2016), used in 6 studies [ICD-8 & ICD-10 (Johannsen et al., 2020); 4 ICD-9 (Schiff et al., 2006; Comtois et al., 2008; Weng et al., 2016, 2018); 1 ICD-10 (Vigod et al., 2019)]; 2 studies used medical records (Taylor et al., 2016; Gressier et al., 2017), 1 study measured SA with the Composite International Diagnostic Interview (CIDI) (Belete et al., 2021) and in Morgan et al. study (1997) the measure is not described. Most of the studies assessed SA during postpartum period (*n* = 7), 2 studies during pregnancy and 1 in both periods. All studies achieved scores of 6 or higher on the Newcastle Otawa Scale, indicating an overall good/high quality.

### Association between Perinatal Suicidal Attempts and Risk Factors: Narrative Synthesis

All studies found statistically significant associations in regression models between perinatal SA and other variables, thus concluding that they should be considered risk factors (Table 2). These factors could be categorized as sociodemographic, clinical, obstetric, neonatal, and psychosocial factors.

**Table 2:** Independent risks factors for SA of the studies included in the systematic review (narrative synthesis)

Author, year	Assessement Time	Independent Risk Factors	OR (95%CI)
Morgan et al., 1997	Pregnancy	Women admitted for miscarriage: -before the event -afterwards the event Induced abortion afterwards the event	2.84 (1.67–4.81)*** 2.29 (1.13–4.65)* 3.25 (1.79–5.91)***
Comtois et al., 2008	Postpartum	Prior hospitalization with psychiatric diagnosis Prior hospitalization substance abuse Prior hospitalization dual diagnosis	27.4 (10.6–70.8)* 6.2 (2.8–13.9)* 11.1 (5.1–24.2)*
Taylor et al., 2016	Pregnancy	Tobacco use in pregnancy Self-harm in previous 2 years	3.64 (1.30-10.19)* 2.55 (1.05–6.50)*
Vigod et al., 2019	Postpartum	Number of previous births Prior maternal psychiatric diagnosis within 2 years before the index birth	1.44 (1.39–1.51)* 7.01 (6.25–7.86)*
Gressier et al., 2017	Pregnancy	Alcohol use in severe mental illness Smoking in severe mental illness Miscarriage	2.37 [1.02–5.53)** 1.87 (1.01–3.49)** 2.29 (1.18–4.41)**
	Postpartum	Maternal younger age Current major depressive disorder Recurrent depression	0.96 (0.93-0.096)* 2.72 (1.40–5.26)** 4.12 (2.25–7.51)***
Weng et al., 2016	Postpartum	Never having married Widowed/Divorced the year before delivery Caesarean delivery Suicide history before delivery Postpartum depressive disorder	2.06 (1.09–3.88)* 5.06 (2.15–11.93)*** 2.38 (1.56–3.62) *** 32.34 (3.52-297.11)** 20.27 (8.99–45.73)***
Weng et al., 2018, 2016	Postpartum	Marital Status: Others(A) Miscarriage (including stillbirth) Termination of pregnancy Mood disorder Bipolar disorder Anxiety disorder Suicidal behavior before	3.24 (2.33–4.49)*** 2.1 (1.66–2.65)*** 2.5 (1.63–3.82)*** 10.88 (5.53–21.80)*** 14.20 (4.02–50.11)*** 4.10 (2.30–7.30)*** 22.07 (8.20–59.40)***
Schiff et al., 2006	Postpartum (1 year)	Fetal and infant death Two previous deliveries Three or more previous deliveries Smoking during pregnancy	3.1 (1.4–7.3)* 1.8 (1.4–2.4)* 3.1 (2.4–4.2)* 2.7 (2.2–3.4)*
Johannsen et al., 2020	Postpartum	Postpartum mental disorders first onset vs. mother population Mental disorders outside postpartum period of 90 days First 12 months after PPMD diagnosis vs. after	6.2 (4.9-8.0)**+** 10.1 (9.6–10.5)**+** 13.5 (8.4–21.7)**+**

*p≤ .05; **p ≤ .005; ***p≤ .0001

+ Hazard Ratio (Cox regression)

A = “Others” exclude being married(reference) and not being married

PPMD = Postpartum Mental Disorder

Sociodemographic variables were the most studied variables. Younger age was found to increase the risk of SA in several studies (Comtois et al., 2008; Taylor et al., 2016; Gressier et al., 2017; Vigod et al., 2019). Having a lower education level (Weng et al., 2018) and marital status (such as being unmarried, widow or divorced) were associated with perinatal SA in some studies (Weng et al., 2016, 2018), but not in others (Gressier et al., 2017). Additionally, not having a partner during pregnancy was not found to be a risk factor for SA in women with severe mental illness (Taylor et al., 2016). Other sociodemographic variables, such as the role of insurance status increased the risk of postpartum SA (Comtois et al., 2008) whereas mother income (Weng et al., 2016, 2018), employment (Gressier et al., 2017), immigrant status (Comtois et al., 2008) or urbanization of residence area (Weng et al., 2018) did not increase the risk for perinatal SA.

Clinical variables such as psychiatric disorders and suicidal behaviors have been identified. Gressier et al. (2017) did not find differences in history of mental illness between women with and without perinatal SA. However, having a prior psychiatric diagnosis within 2 years before the index birth was found to increase the risk of self-inflicted injury (self-harm or suicide attempts) in postpartum (Vigod et al., 2019). Having a current psychiatric diagnosis increased the risk of postpartum SA resulting in hospitalization (Comtois et al., 2008). Moreover, women with first onset postpartum mental disorders had a higher risk of self-harm compared to mothers without mental disorders (Johannsen et al., 2020). Mood disorders were associated with postpartum suicide attempts in most studies. Having a mood disorder or a specific diagnosis of postpartum depression was found to increase the risk of SA in postpartum women (Weng et al., 2016, 2018). Similar results were obtained for major depression or recurrent depression but not for bipolar disorders or chronic depression (Gressier et al., 2017). The results of other psychopathological problems such as substance use disorders (Comtois et al., 2008; Taylor et al., 2016; Gressier et al., 2017; Weng et al., 2018), anxiety, psychosis or bipolar disorder as risk factors were contradictory (Gressier et al., 2017; Weng et al., 2018). Some additional clinical variables, such as level of functioning, acute admissions in the 2 years preceding pregnancy, psychotropic drugs used or stopping medication were considered as risk factors for SA in pregnant women with severe mental illness, but these variables were not found to be independent risk factors in regression models. Only self-harm within 2 years before pregnancy was identified as a risk factor (Taylor et al., 2016). Similarly, history of suicide attempts was also found to be a risk factor of postpartum SA in perinatal women from general population (Weng et al., 2016, 2018).

Obstetric variables are relevant in the study of suicidal attempts in perinatal period. Number of previous births was found to increase the risk of postpartum self-inflicted injury and SA (Vigod et al., 2019). However, other studies did not find significant differences in parity (Schiff et al., 2006; Comtois et al., 2008) or gravidity (Comtois et al., 2008) in the final multivariate models. Birth method was also studied. Weng et al. (2016) identified caesarean delivery as a risk factor for attempted suicide within 1 year postpartum. However, other studies did not find this association (Schiff et al., 2006) even if it was an emergency caesarean (Gressier et al., 2017). Difficulty in conceiving (Gressier et al., 2017), unwanted pregnancy (Belete et al., 2021), infant sex (Weng et al., 2016; Gressier et al., 2017), multiple gestation or inadequate obstetric monitoring (Gressier et al., 2017) were not associated with SA in this period. Complications of pregnancy or of labor and delivery were also not related to SA (Schiff et al., 2006; Gressier et al., 2017). Preeclampsia specifically was not associated with SA in either pregnant or postpartum women(Weng et al., 2016; Gressier et al., 2017). Regarding adverse infant outcomes, prematurity (Schiff et al., 2006), newborn low birth weight (Schiff et al., 2006; Weng et al., 2016; Gressier et al., 2017) and the presence of congenital malformations (Schiff et al., 2006) was not found to be a risk factor of SA during pregnancy or the postpartum period. Perinatal loss was studied by several researchers. The risk of SA was higher in women who experienced miscarriage or voluntary termination of pregnancy then those who had a live birth (Morgan et al., 1997; Weng et al., 2018). Similarly, Gressier et al. (2017) identified history of miscarriage as a risk factor for SA in pregnancy. However, history of abortion was not found to be associated. Fetal death or the death of an infant in the first year after delivery was identified as a risk factor for a postpartum SA hospitalization (Schiff et al., 2006). Finally, SA in pregnancy were associated with alcohol use and smoking during pregnancy in women with severe mental illness (Gressier et al., 2017). Similar associations were found for tobacco use and SA during pregnancy (Taylor et al., 2016) and for postpartum SA (Schiff et al. 2006) in women with mental disorders.

Significant themes to emerge in terms of psychosocial risk factors for SA were childhood abuse and domestic violence. Taylor et al. (2016) found that self-harm in pregnancy was associated with a history of child abuse and past/current domestic violence, but these findings were not statistically significant in multivariable analysis. Gressier et al. (2017) did not find differences in foster care in childhood, maltreatment in childhood or sexual abuse between women with and without suicidal attempts in pregnancy/postpartum. Additionally, poor family/social support (Gressier et al., 2017) has not been associated with SA in mothers with mental illness. No differences were found across different groups for the support from the child’s father.

### Meta-Analysis Results

All analyses performed are detailed below, grouped according to pre-established categories, and stratified by assessment period and/or type of sample. The forest plot figures can be found in the supplementary material (Figures S1-S12).

### Sociodemographic Variables

The sociodemographic variables studied in 3 or more studies were marital status, education level, age and race (Figures S1-S4). Marital status was assessed in 5 studies, and for this meta-analysis it was dichotomized into married/not married. In all 5 studies marital status was assessed in the postpartum period while in one it was also assessed in pregnancy (Gressier et al., 2017), so only the effect of the variable in the postpartum period has been synthesised. This is true for the majority of studies described subsequently. The risk of SA in the postpartum period was higher in not married women compared to married women (pooled OR = 1.87, 95% CI = 1.26–2.78). There was high heterogeneity between studies (*I*^2^ = 91.2%). There were differences in effect size by type of sample (Q(1) = 21.8, *p* < .001). Compared to the community sample, in the clinical sample the risk of postpartum SA was higher in married than in not married women. Education level was assessed also in 5 studies, and it was dichotomized into higher education /not higher education. The risk of SA in the postpartum period was higher in women with no higher education compared to women with higher education (pooled OR = 1.89, 95% CI = 1.31–2.74). There was high heterogeneity between studies (*I*^2^ = 85.1%). There were differences in effect size by type of sample (Q(1) = 25.4, *p* < .001). In the community sample women with no higher education had higher risk of postpartum SA compared to those with higher education. However, this association was not observed in the clinical sample. Age (older / not older age) was not associated with risk of SA in postpartum. All studies that included the variable age were conducted in community sample. Heterogeneity between studies was high (*I*^2^ = 91%). Race (White / not White) was measured in 3 studies, two of them in community sample and measuring SA in postpartum and one of them (Taylor et al., 2016) in clinical sample and measuring SA in pregnancy. Race was not associated with risk of perinatal SA. Heterogeneity between studies was low (*I*^2^ = 19.2%).

### Clinical Variables

The clinical variables studied in 3 or more studies were mood disorder, anxiety disorder and previous suicidal behaviour (Figures S5-S8). Studies including mood disorder have assessed SA in pregnancy (3 studies: Gressier et al., 2017; Weng et al., 2016, 2018) and in postpartum (2 studies: Gressier et al., 2017; Taylor et al., 2016). To avoid a unit of analysis error, a separate analysis of SA in postpartum and SA in pregnancy has been performed. The risk of SA in the postpartum period was higher in women with mood disorder compared to women without mood disorder (pooled OR = 11.43, 95% CI = 1.56–83.87). The heterogeneity between studies was high (*I*^2^ = 97%). Effect sizes were higher in community than in clinical samples (Q(1) = 65.4, *p* < .001). However, mood disorder was not associated with higher risk of SA in pregnancy. In this case, all studies were conducted in clinical samples.

Studies involving anxiety disorder have evaluated postpartum SA. Anxiety disorder was not associated with higher risk of postpartum SA. Heterogeneity between studies was high (*I*^2^ = 89.3%). There were differences in effect size between community and clinical samples (Q(1) = 10.8, *p* < .001). In community samples anxiety disorder was associated with higher risk of postpartum SA (pooled OR = 7.45, 95% CI = 3.61–15.36), while in clinical samples this relationship disappeared.

Suicidal behavior before was assessed in 3 studies, 2 studies assessed postpartum SA in community sample, and 1 study assessed SA in pregnancy in clinical sample (Taylor et al., 2016). The risk of SA in the perinatal period was higher in women with previous suicidal behavior, although the pooled OR obtained a very wide confidence interval (pooled OR = 38.04, 95% CI = 3.36–431.17). There was high heterogeneity between studies (*I*^2^ = 81%). Effect sizes were higher in community than in clinical samples (Q(1) = 10.2, *p* < .001).

### Obstetric and Neonatal Variables

The obstetric and neonatal variables studied in 3 or more studies was newborn low birth weight, smoking during pregnancy and caesarean section (Figures S9-S2). The variable perinatal loss appears in the different studies defined in a very heterogeneous way (miscarriage, history of miscarriage, fetal and infant death), so it has not been possible to synthesize quantitatively.

Newborn low birth weight and caesarean section (CS) were measured in 3 studies assessing postpartum SA, 2 studies in community sample and 1 study in clinical sample. Newborn low birth weight was not associated with higher risk of postpartum SA. There was high heterogeneity between studies (*I*^2^ = 77.9%). There were differences in effect sizes by sample type (Q(1) = 8.7, *p* < .001). Newborn low birth weight was associated with higher risk of postpartum SA in the community sample but not in the clinical sample. Caesarean section was not associated with higher risk of postpartum SA. There was high heterogeneity between studies (*I*^2^ = 92.6%).

Finally, smoking during pregnancy was measured in 2 studies assessing postpartum SA and 2 studies assessing SA in pregnancy. To avoid a unit of analysis error, pregnancy and postpartum were analysed separately. Smoking during pregnancy was not associated with postpartum SA. There was high heterogeneity between studies (*I*^2^ = 96.6%). In contrast, smoking during pregnancy was associated with higher risk of SA in pregnancy (pooled OR = 3.87, 95% CI = 1.35–11.11). There was high heterogeneity between studies (*I*^2^ = 80.2%).

## Discussion

This study aimed to provide a systematic overview of all risk factors pertaining to perinatal SA in the literature. To our knowledge, this is the first meta-analysis of risk factors of perinatal SA. Our results show that unmarried women, with no higher education and affected by a mood disorder have a higher risk of postpartum SA; women who smoke during pregnancy have a higher risk of SA in pregnancy; and women with previous suicidal behavior have a higher risk of perinatal SA, whether during pregnancy or in the postpartum period. The type of sample, whether community or clinical, is a relevant moderating factor.

Although there are not meta-analysis or systematic reviews carried out exclusively in female samples and/or perinatal period, the variables that we found associated with SA in our results are consistent with those found in previous systematic reviews and metanalysis carried out in mixed samples (men and women) (Li et al., 2012; Teti et al., 2014; Echeverria et al., 2021), and in samples of men (Richardson et al., 2021). Having major depression and previous suicide attempts (along with family disfunction) are strong risk factors for suicidal behavior in Teti’s et al. (2014) revision. Not being married and having a diagnosis of depression were found to be strong risk factors associated with suicidal behavior in Richardson’s et al. (2021) systematic review, along with alcohol and drug use/dependence. In this narrative review, low levels of education and tobacco use were also found to be associated with suicidal behavior in men. Several metanalysis have shown an association between tobacco use and suicidality (Li et al., 2012; Echeverria et al., 2021). Furthermore, the WHO (2014) reported that a prior attempted suicide is the largest risk factor for suicide in the general population. Oates (2003) reported that among the maternal deaths caused by suicide in the United Kingdom from 1997 to 1999, a significant proportion of the women had a psychiatric disorder. Finally, several studies also have found an association between suicidality and not been married and low education in women (Bálint et al., 2016; Øien-Ødegaard et al., 2021; Choi et al., 2022).

However, in our results we have not found a relationship between suicide attempts and other variables consistently associated with suicidal behavior or attempts, such as traumatic events in childhood or childhood adversity (Angelakis et al., 2020; Grummitt et al., 2021; Xiao et al., 2023) or intimate partner violence (Devries et al., 2013). This is probably because most studies have not included these variables. Only two studies carried out in clinical samples included these variables (Taylor et al., 2016; Gressier et al., 2017), although they found no association with SA in multivariate analysis.

Regarding the effect of specifically perinatal factors, it has only been possible to meta-analyze two obstetric variables, newborn low birth weight and cesarean section, the rest of the variables were evaluated in few studies or in a very heterogeneous way. These two variables were not found to be associated with SA. Perinatal loss is the variable that appears to be most consistently associated with SA, although it could not meta-analiyzed due to its varied definition across studies. Finally, we want to draw attention to the scarcity of studies evaluating factors such as unplanned pregnancy or premenstrual symptoms. Especially premenstrual symptoms, since they have been associated with a higher risk of suicide attempt in a meta-analysis of 10 studies (Prasad et al., 2021).

To our knowledge, this is the first systematic review to analyze risk factors for SA during pregnancy and postpartum, distinguishing between perinatal SA from other related outcomes (suicidality, suicidal ideation, suicidal risk). This is also the first meta-analysis to measure the effect of some sociodemographic, obstetric, neonatal and clinical variables as risk factors for perinatal SA. Search terms were carefully selected to capture all possible studies in different indexed databases. The participation of independent reviewers helped ensure the validity of our results.

However, several limitations should be considered. First, only 10 studies were finally included in the present review. Although no limitation by year was used, many studies were excluded because they did not distinguish between SA and suicidal ideation, risk of suicide or even completed suicide. Second, heterogeneity in the studies was observed due to the various definitions used for assessing SA. The measure most used was the ICD (ICD-8, ICD-9 and ICD-10 codes). ICD codes are unable to determine suicidal intent but do allow to identify intentionality in self-injurious behaviors. But in several studies, a general spectrum of SA has been applied, also capturing accidental injuries (Vigod et al., 2019; Johannsen et al., 2020). Even when codes related to self-injurious behaviors of undetermined intent were not included, it is not possible to distinguish between injuries where a women intended to die versus self-harm, such as for self-regulation or a plea for help (Schiff et al., 2006; Comtois et al., 2008; Weng et al., 2016, 2018). On the other hand, perinatal SAs reported by the patient (Gressier et al., 2017; Belete et al., 2021) or registered in the medical files (Taylor et al., 2016; Gressier et al., 2017) could also be underestimated, especially because of socially desirability bias and worries about custody loss (Dolman et al., 2013). Third, although a wide variety of variables were explored in the included studies, most of them were risk factors related to the general population. It would be necessary to include gender related risk factors for SA, and specific variables related to reproductive life events. Forth, studies about SA during pregnancy and conducted in clinical populations were unrepresented. Fifth, due to the lack of studies our meta-analysis focused only on some risk factors, so but it would be interesting to conduct meta-analyses on other risk factors when data become available. Finally, several pooled effect estimates were considerably heterogeneous, suggesting significant between-study variability so the aggregate findings reported in this paper need to be treated with caution.

### Implications for Practice and/or Policy

Understanding the risk factors associated with suicidal behavior during pregnancy and the postpartum period is crucial for early detection and intervention. Although the prevalence of SA is not higher in pregnant and postpartum women compared to 1-year prevalence of SA in other populations (Rao et al., 2021), the negative impact of SA on health outcomes and potential loss of life must be seriously considered. To screen for the risk factors found as part of routine perinatal care would facilitate early identification and support for at-risk individuals, enabling healthcare professionals to offer targeted interventions such as counseling, social support programs, and smoking cessation assistance. From a policy perspective, the findings suggest a pressing need for the development and implementation of comprehensive perinatal mental health programs that not only aim to support mental health but also address social determinants of health, like education and partner support.

## Conclusion

In conclusion, our study extends prior reviews about suicidal behaviors by studying in women perinatal suicide attempts independently, as well as it synthesized data on some sociodemographic, clinical, obstetric and neonatal risk factors. Although some factors are relevant for general population, identifying risk factors in perinatal women provides a step forward towards understanding suicide attempts during pregnancy and postpartum.

Further studies are needed to identify specific risk factors for perinatal suicidal behaviour to improve detection of women at risk, in whom early intervention may be of benefit.

### Electronic supplementary material

Below is the link to the electronic supplementary material.

Supplementary Material 1
