# Supplementary material for: Suicide Attempts during Pregnancy and Postpartum: A Systematic Review and Meta-Analysis
Source: Matern Child Health J. 2024 Jun 29;28(9):1443–53. doi: 10.1007/s10995-024-03956-w (PMC11358321; doi:10.1007/s10995-024-03956-w)
Supplement: Supplementary file 1 — Supplementary Material 1 [file 10995_2024_3956_MOESM1_ESM.docx]

**Figure S1**

*Forest plot summarizing the effect of marital status on risk of postpartum suicide attempts*


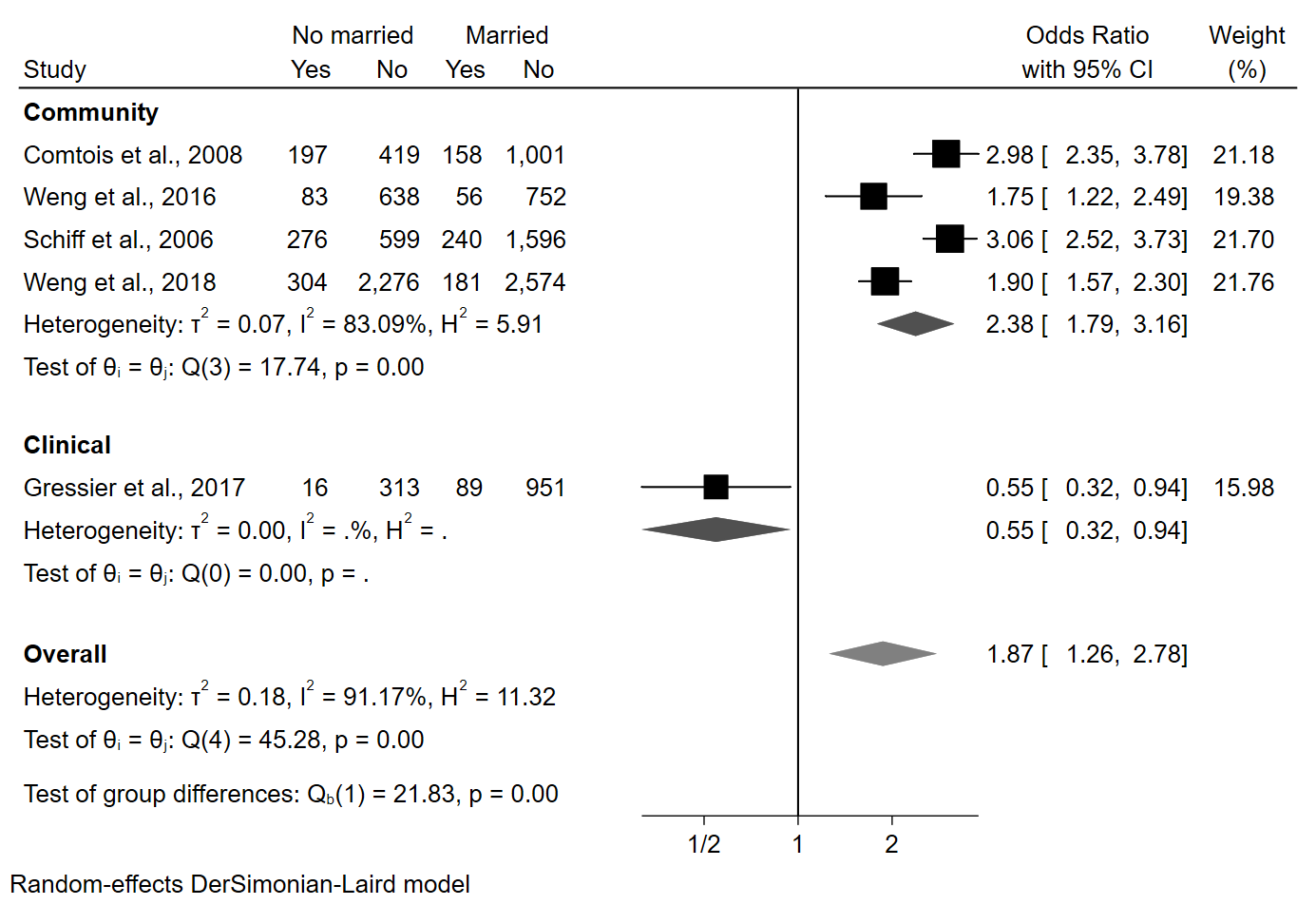


*Note.* CI = confidence interval; τ^2^ = tau-squared statistic; I^2^ = I-squared statistic; H^2^ = H-squared statistic; θ = estimated parameter; θi = parameter of ith study; θj = parameter of jth study; Q = Cochran Q statistic; p = significance value.

**Figure S2**

*Forest plot summarizing the effect of education level on risk of postpartum suicide attempts*


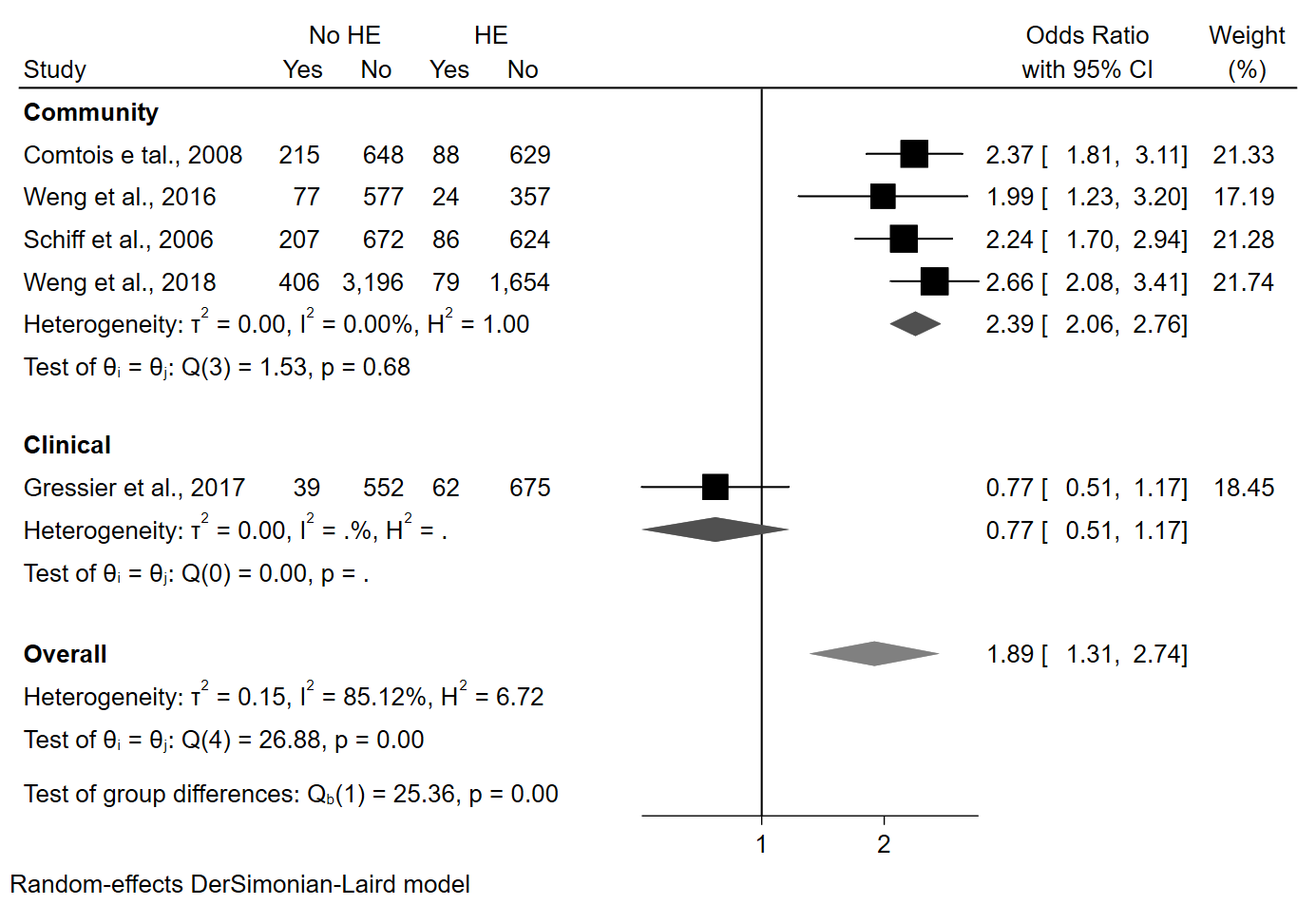


*Note.* HE = higher education; CI = confidence interval; τ^2^ = tau-squared statistic; I^2^ = I-squared statistic; H^2^ = H-squared statistic; θ = estimated parameter; θi = parameter of ith study; θj = parameter of jth study; Q = Cochran Q statistic; p = significance value.

**Figure S3**

*Forest plot summarizing the effect of age on risk of postpartum suicide attempts*


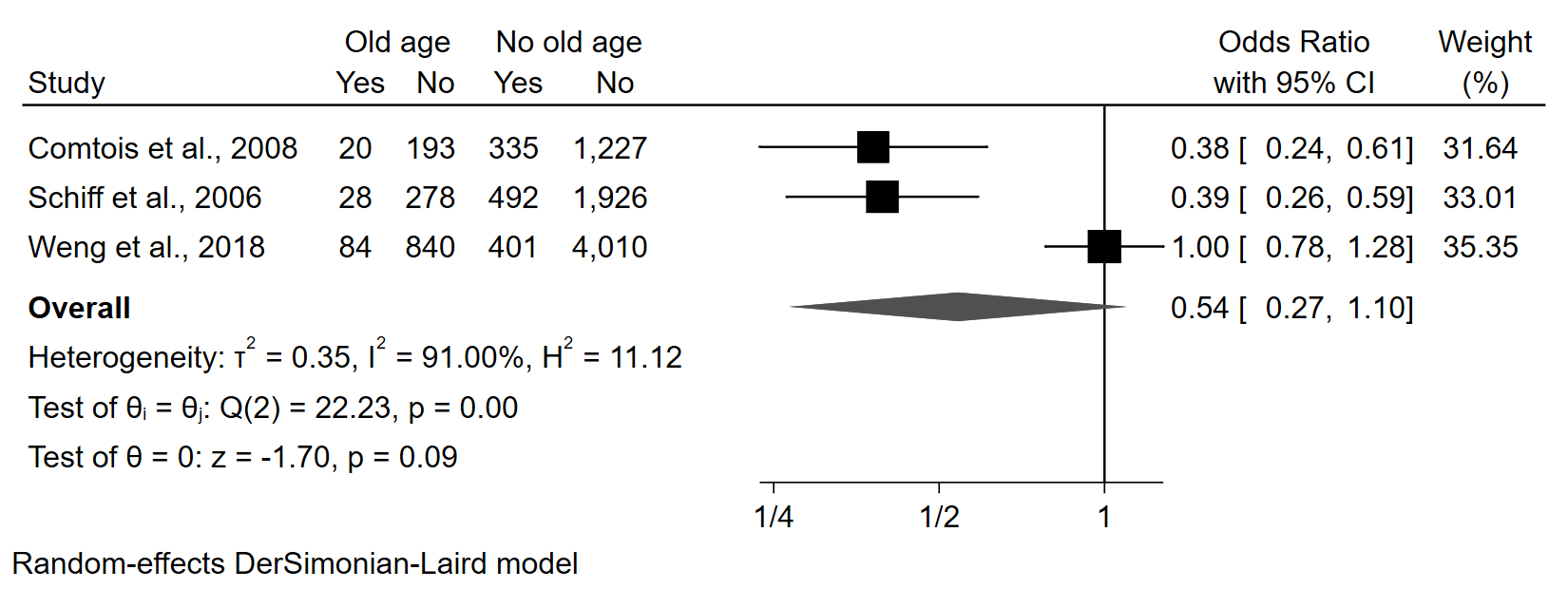


*Note.* CI = confidence interval; τ^2^ = tau-squared statistic; I^2^ = I-squared statistic; H^2^ = H-squared statistic; θ = estimated parameter; θi = parameter of ith study; θj = parameter of jth study; Q = Cochran Q statistic; p = significance value.

**Figure S4**

*Forest plot summarizing the effect of race on perinatal suicide attempts*


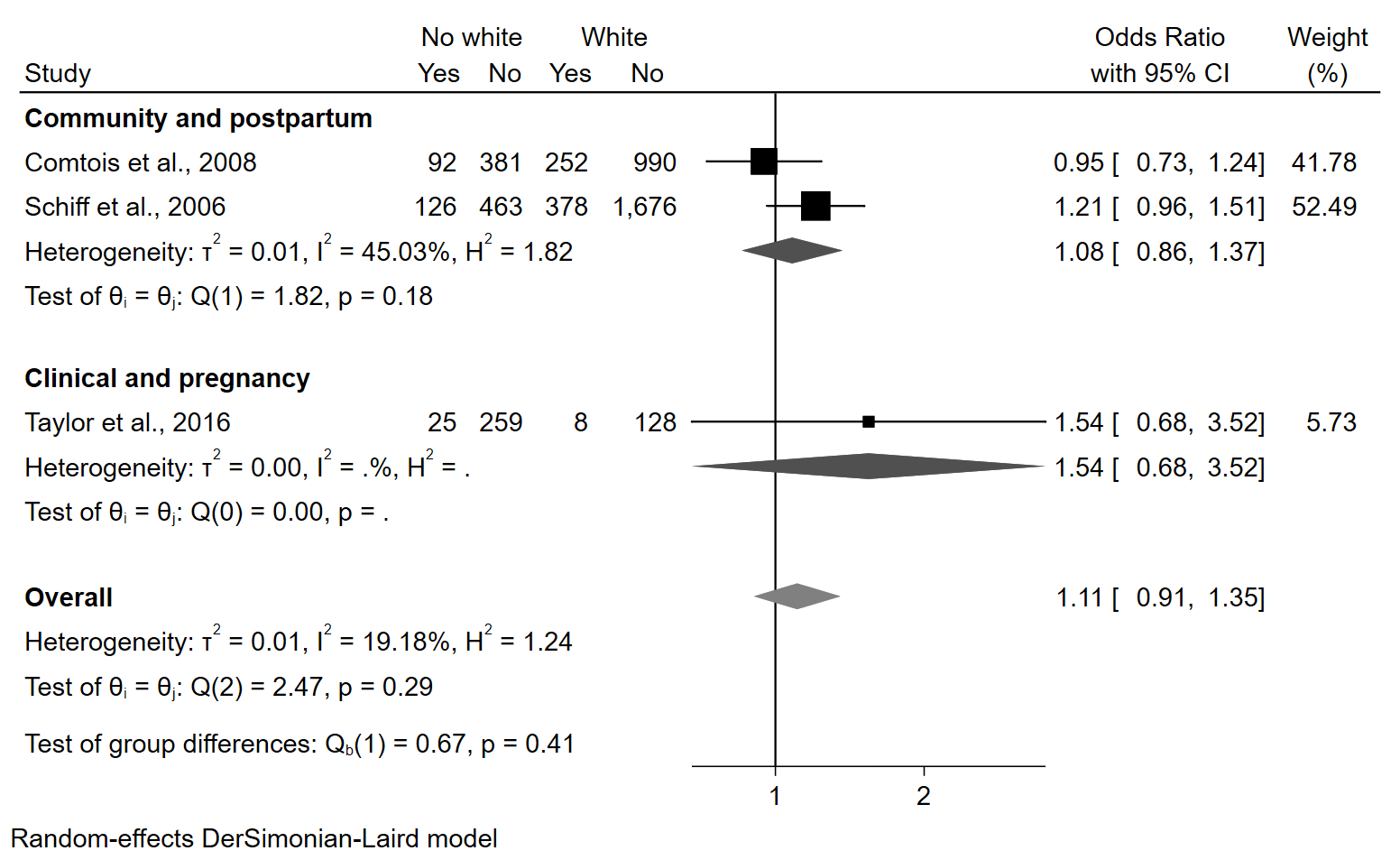


*Note.* CI = confidence interval; τ^2^ = tau-squared statistic; I^2^ = I-squared statistic; H^2^ = H-squared statistic; θ = estimated parameter; θi = parameter of ith study; θj = parameter of jth study; Q = Cochran Q statistic; p = significance value.

**Figure S5**

*Forest plot summarizing the effect of mood disorder on postpartum suicide attempts*


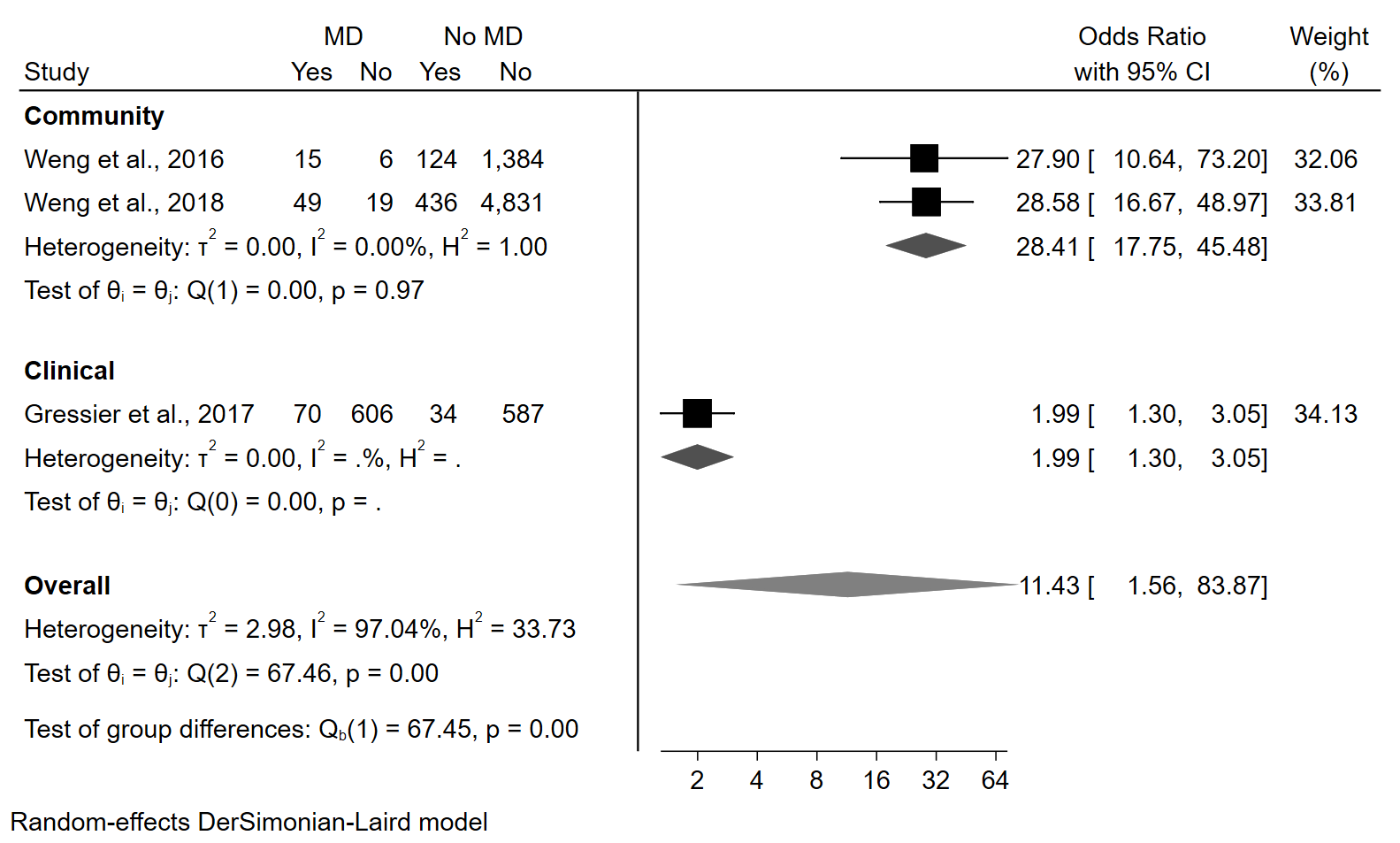


*Note.* MD = mood disorder; CI = confidence interval; τ^2^ = tau-squared statistic; I^2^ = I-squared statistic; H^2^ = H-squared statistic; θ = estimated parameter; θi = parameter of ith study; θj = parameter of jth study; Q = Cochran Q statistic; p = significance value.

**Figure S6**

*Forest plot summarizing the effect of mood disorder on suicide attempts in pregnancy*


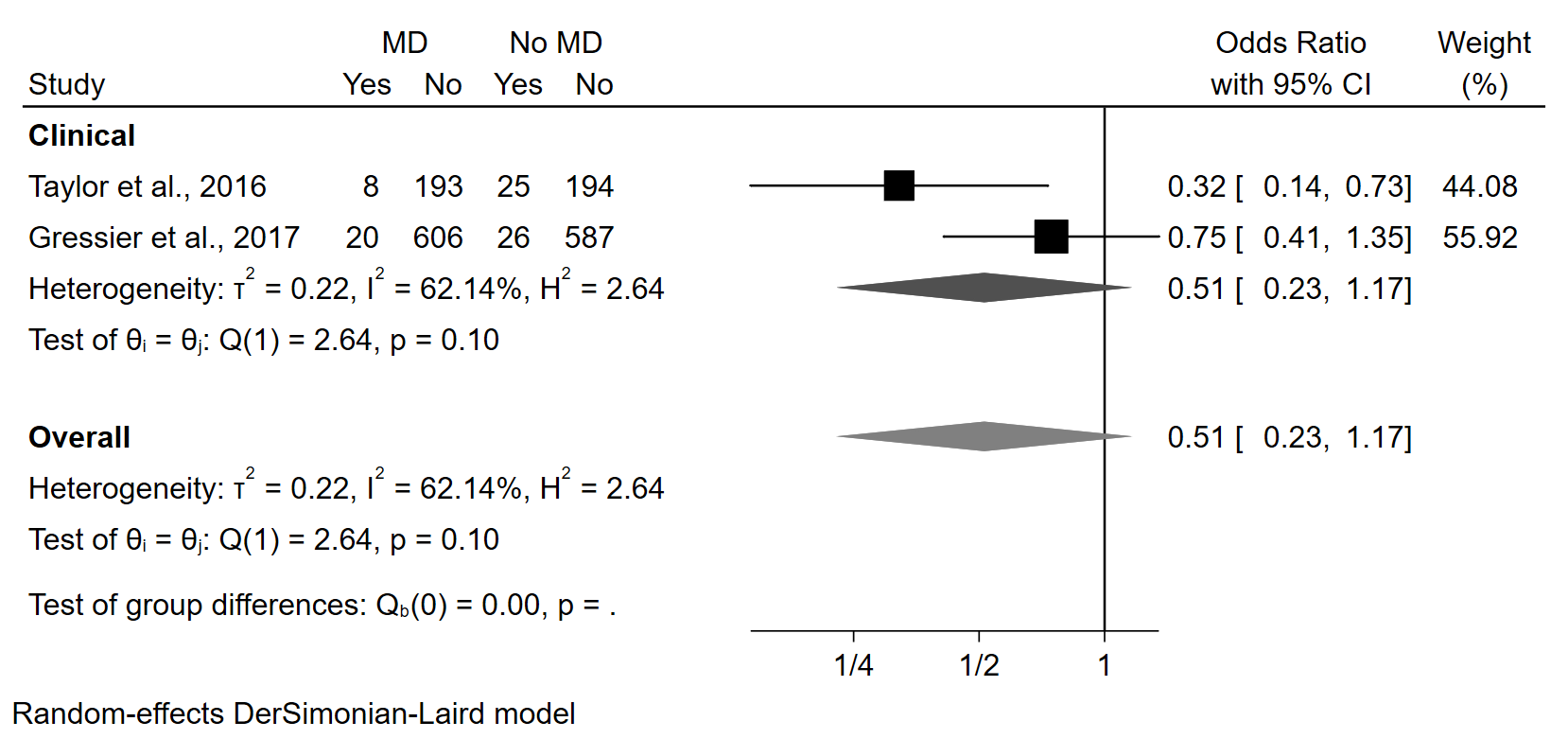


*Note.* MD = mood disorder; CI = confidence interval; τ^2^ = tau-squared statistic; I^2^ = I-squared statistic; H^2^ = H-squared statistic; θ = estimated parameter; θi = parameter of ith study; θj = parameter of jth study; Q = Cochran Q statistic; p = significance value.

**Figure S7**

*Forest plot summarizing the effect of anxiety disorder on postpartum suicide attempts*


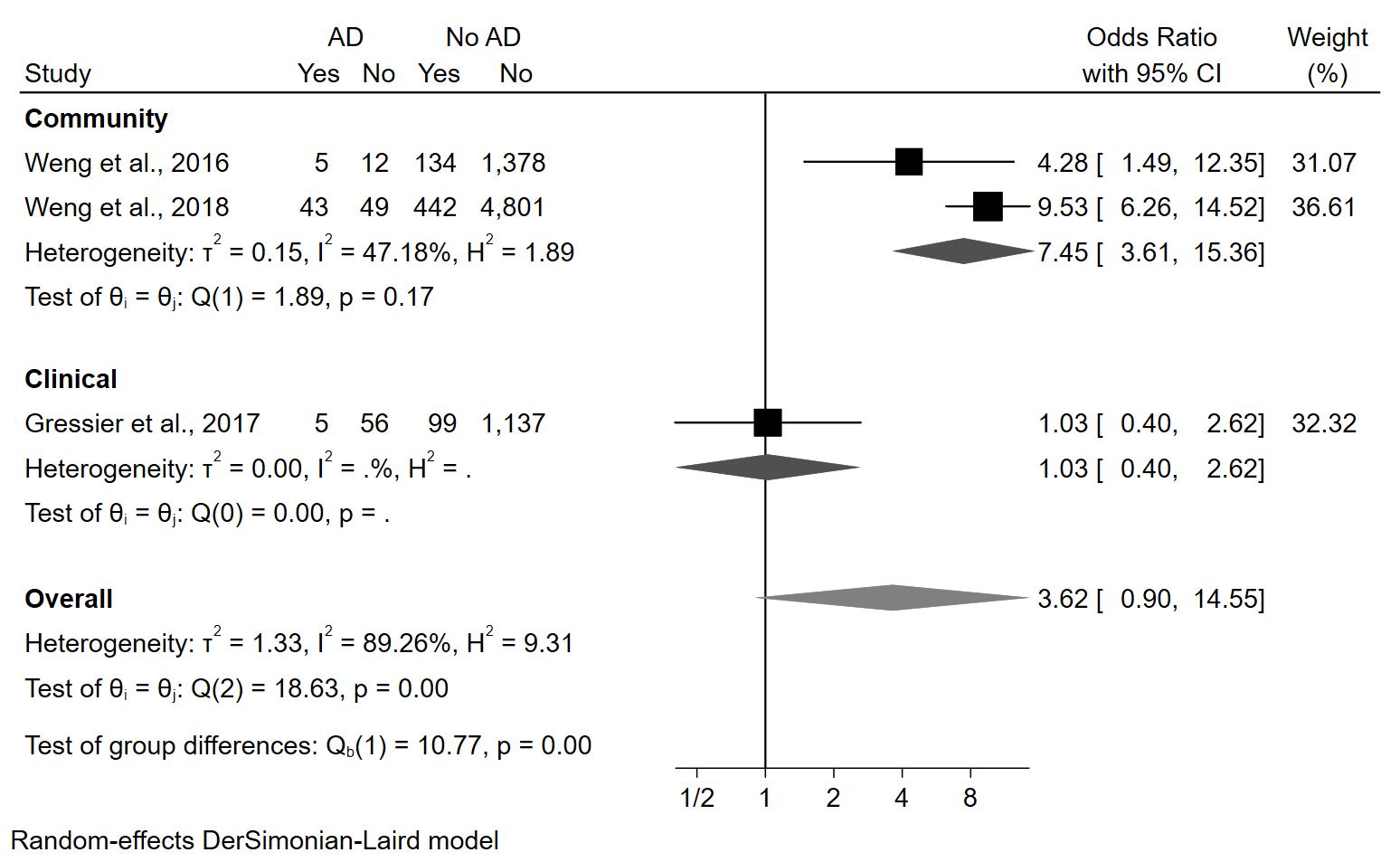


*Note.* AD = anxiety disorder; CI = confidence interval; τ^2^ = tau-squared statistic; I^2^ = I-squared statistic; H^2^ = H-squared statistic; θ = estimated parameter; θi = parameter of ith study; θj = parameter of jth study; Q = Cochran Q statistic; p = significance value.

**Figure S8**

*Forest plot summarizing the effect of suicidal behavior before on perinatal suicide attempts*


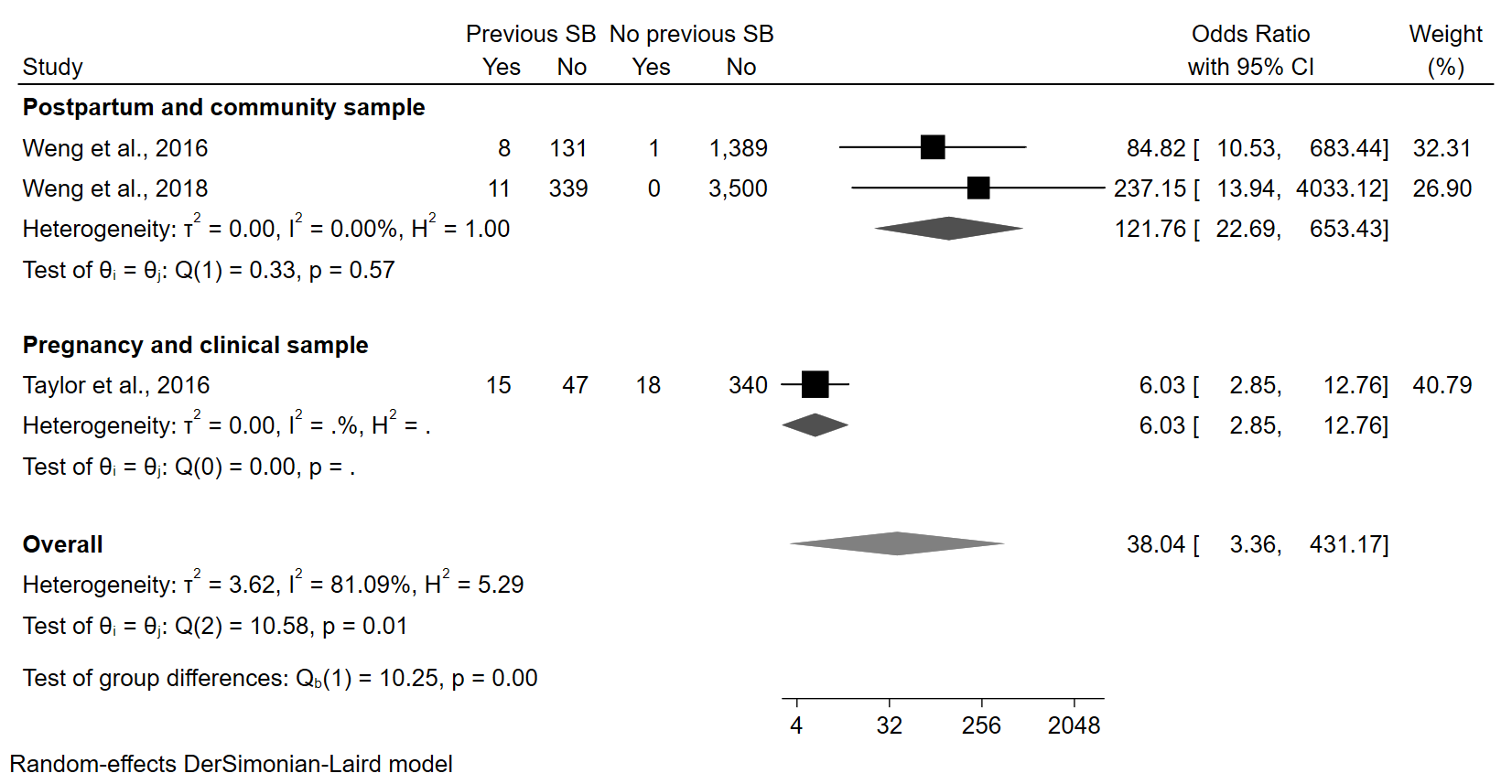


*Note.* SB = suicidal behavior; CI = confidence interval; τ^2^ = tau-squared statistic; I^2^ = I-squared statistic; H^2^ = H-squared statistic; θ = estimated parameter; θi = parameter of ith study; θj = parameter of jth study; Q = Cochran Q statistic; p = significance value.

**Figure S9**

*Forest plot summarizing the effect of newborn low birth weight on postpartum suicide attempts*


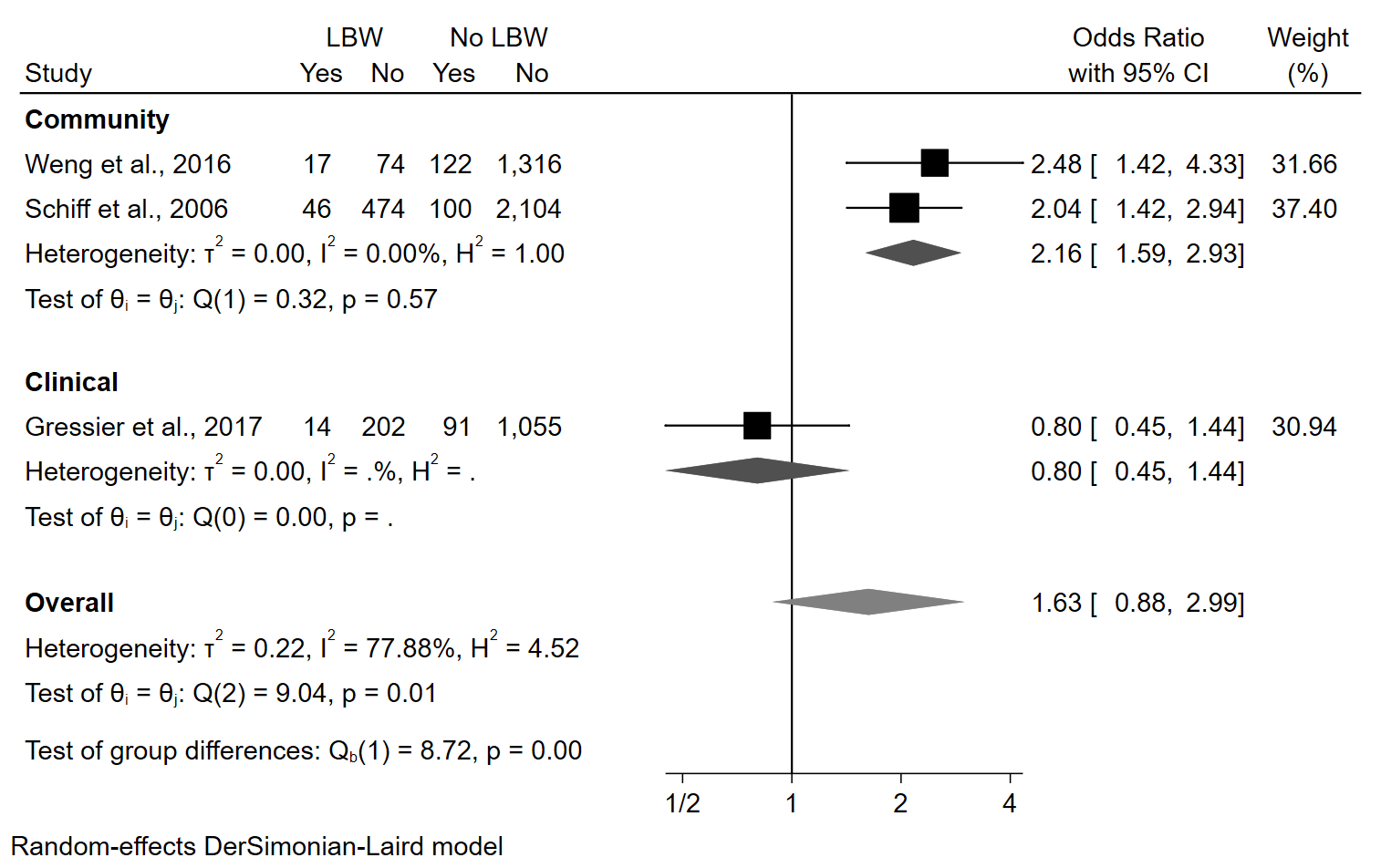


*Note.* LBW = newborn low birth weight; CI = confidence interval; τ^2^ = tau-squared statistic; I^2^ = I-squared statistic; H^2^ = H-squared statistic; θ = estimated parameter; θi = parameter of ith study; θj = parameter of jth study; Q = Cochran Q statistic; p = significance value.

**Figure S10**

*Forest plot summarizing the effect of caesarean section on postpartum suicide attempts*


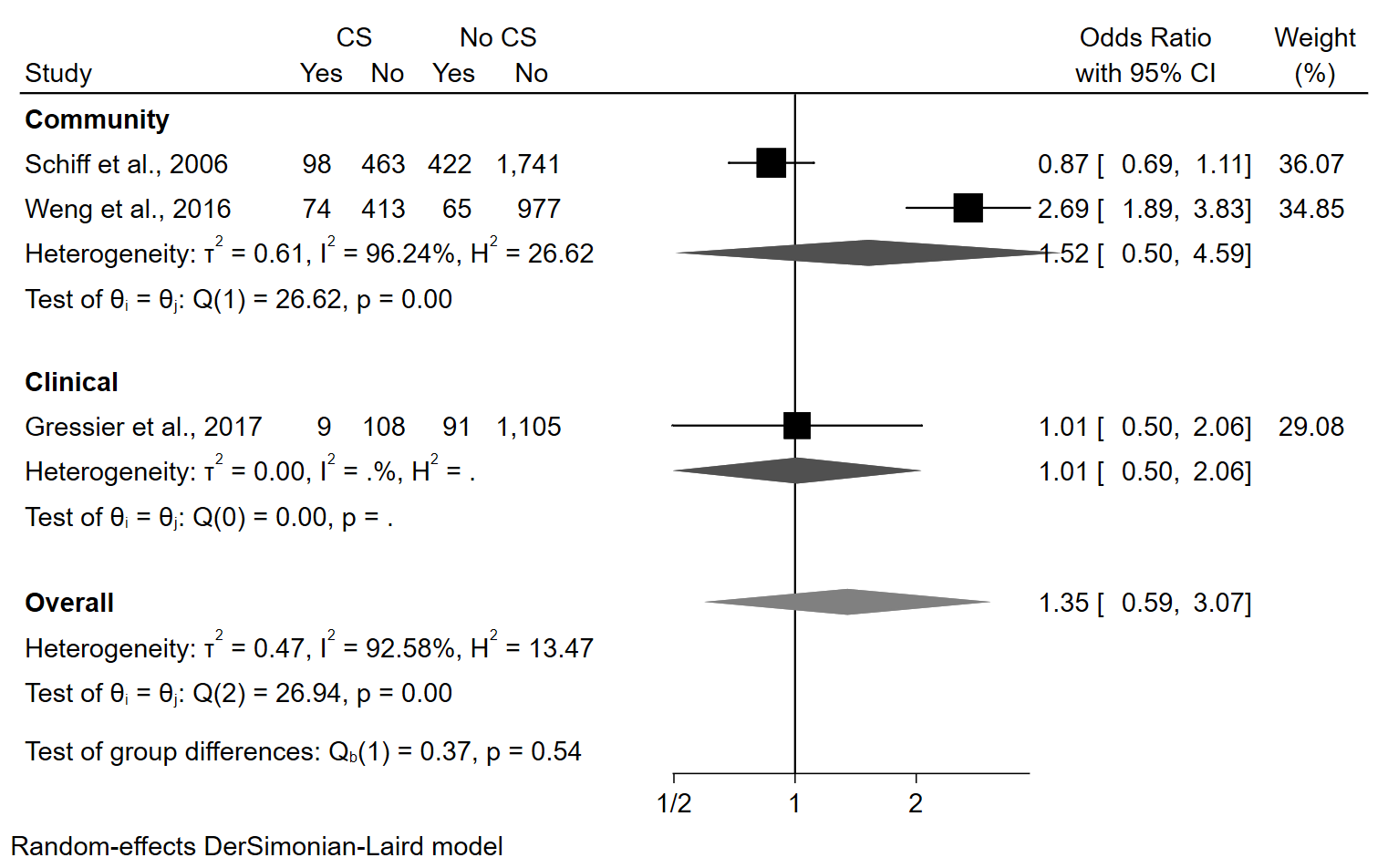


*Note.* CS = caesarean section; CI = confidence interval; τ^2^ = tau-squared statistic; I^2^ = I-squared statistic; H^2^ = H-squared statistic; θ = estimated parameter; θi = parameter of ith study; θj = parameter of jth study; Q = Cochran Q statistic; p = significance value.

**Figure S11**

*Forest plot summarizing the effect of smoking during pregnancy on postpartum suicide attempts*


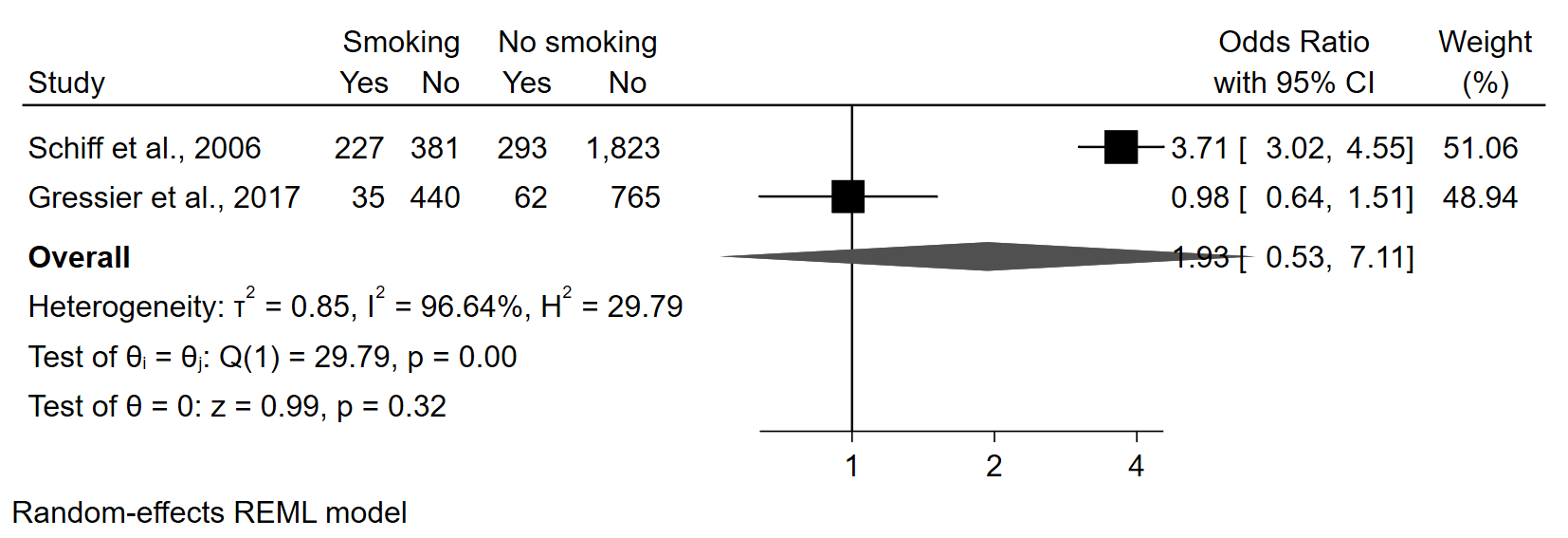


*Note.* CI = confidence interval; τ^2^ = tau-squared statistic; I^2^ = I-squared statistic; H^2^ = H-squared statistic; θ = estimated parameter; θi = parameter of ith study; θj = parameter of jth study; Q = Cochran Q statistic; p = significance value.

**Figure S12**

*Forest plot summarizing the effect of smoking during pregnancy on suicide attempts in pregnancy*


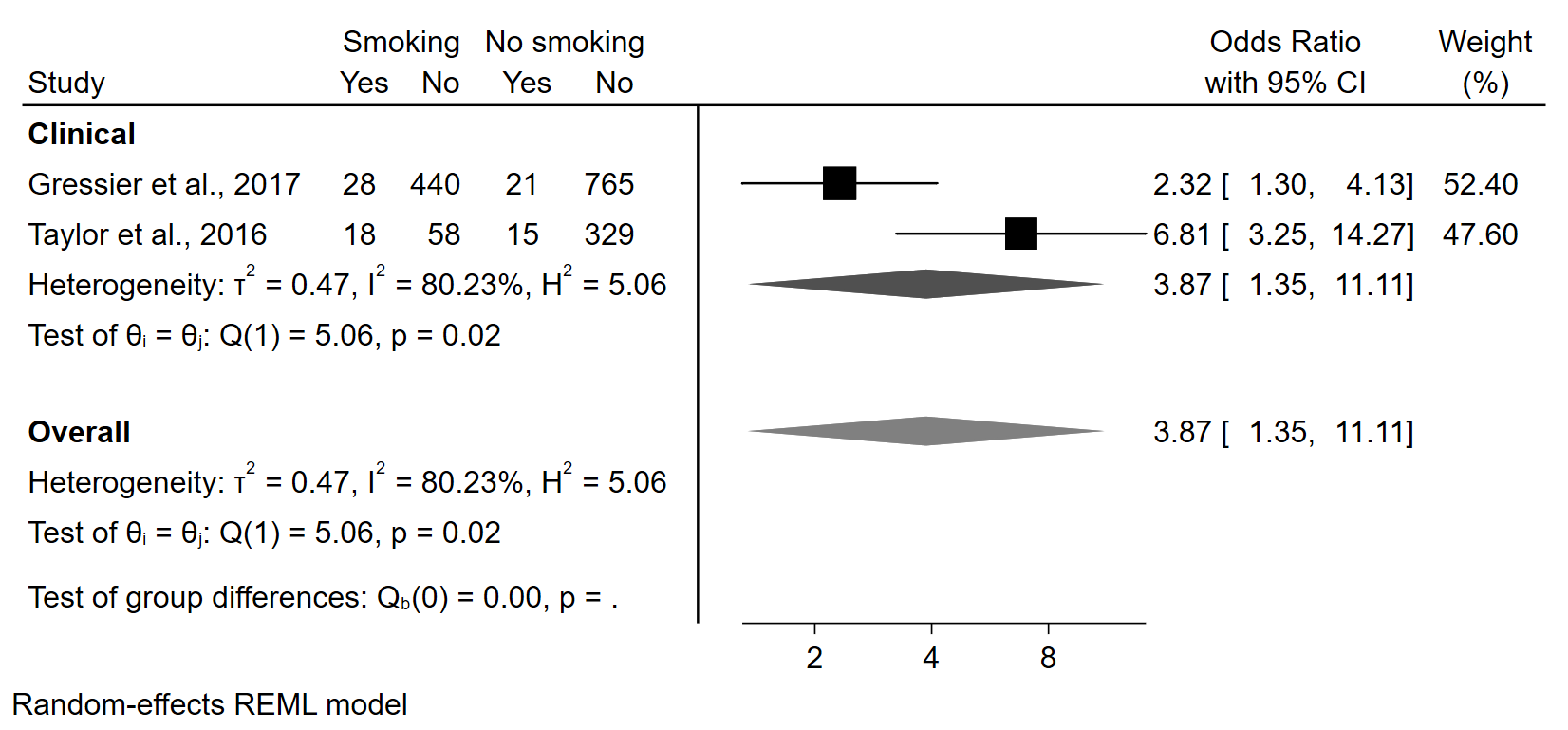


*Note.* CI = confidence interval; τ^2^ = tau-squared statistic; I^2^ = I-squared statistic; H^2^ = H-squared statistic; θ = estimated parameter; θi = parameter of ith study; θj = parameter of jth study; Q = Cochran Q statistic; p = significance value.

Table S1 Summary table of Results

| Category | Risk Factor | Risk of perinatal SA | Studies |
| --- | --- | --- | --- |
| Sociodemographic | Younger age | Increased | Comtois et al. 2008; Taylor et al. 2016; Gressier et al. 2017; Vigod et al. 2019 |
|  | Low educational level | Increased in most studies | Weng et al. 2016, 2018; Gressier et al., 2017 |
|  | Marital status: not married | Increased in most studies | Weng et al. 2016, 2018; Gressier et al., 2017 |
|  | Insurance status | Increased | Comtois et al., 2008 |
|  | Mother income, employment, immigrant status, urbanization | No association | Comtois et al., 2008; Weng et al., 2016; Weng et al., 2018; Gressier et al., 2017 |
| Clinical | History of psychiatric disorder | Controversial | Gressier et al., 2017; Vigod et al., 2019 |
|  | Current psychiatric diagnosis | Increased | Comtois et al., 2008; Johannsen et al., 2020 |
|  | Mood disorder | Increased in the majority of studies | Weng et al. 2016, 2018 |
|  | Substance use disorders, anxiety, psychosis, bipolar disorder | Controversial | Gressier et al. 2017; Weng et al. 2018 |
|  | History of suicide attempts | Increased | Taylor et al., 2016; Weng et al. 2016, 2018 |
| Obstetric | Parity, type of delivery, difficulty in conceiving, infant sex, multiple gestation, inadequate obstetric monitoring, pregnancy/delivery complications | Contradictory or no association | Schiff et al. 2006; Weng et al., 2016; Gressier et al., 2017; Belete et al., 2021 |
|  | Infant adverse outcomes, Newborn low birth weight, congenital malformations | No association | Schiff et al. 2006; Weng et al. 2016; Gressier et al. 2017 |
|  | Perinatal loss | Increased in most studies | Morgan et al. 1997; Weng et al. 2018; Gressier et al., 2017 |
|  | Alcohol and tobacco use during pregnancy | Increased | Gressier et al., 2017; Taylor et al., 2016; Schiff et al., 2006 |
| Psychosocial | Abuse in childhood or adulthood | Controversial or no association | Taylor et al., 2016; Gressier et al., 2017 |
|  | Poor family/social support | No association | Gressier et al., 2017 |
